# Fear of Virus or of Competitors? The Decision Rationales of Financial Managers Under COVID-19

**DOI:** 10.3389/fpsyg.2020.556139

**Published:** 2020-10-27

**Authors:** Jinlu Sun, Ting Wu, Bo Chen

**Affiliations:** ^1^School of Humanities and Social Sciences, Beihang University, Beijing, China; ^2^Chongqing Vocational Institute of Engineering, Chongqing, China; ^3^Institute for Finance and Economics, Central University of Finance and Economics, Beijing, China

**Keywords:** novel coronavirus (COVID-19), financial managers, decision-making, competitors, China

## Abstract

This paper surveyed 422 financial managers before the number of novel coronavirus (COVID-19) infections in China peaked and used path analysis to study the risk decision-making mechanisms of financial managers. The study found that whether financial managers developed coping strategies depends on their assessment of potential business revenue losses. There are two transmission paths: the direct effect refers to the risk perception directly caused by COVID-19, while the indirect effect refers to managers’ fear that they will not make timely adjustments or will make judgment errors, resulting in the loss of competitive advantage. It is worth noting that the indirect effect exceeds the direct effect, which indicates that financial managers are more rational than ordinary people in dealing with COVID-19, that they are relatively more concerned about competitor changes, and that they may even view COVID-19 as an important opportunity to obtain a better competitive position.

## Introduction

The novel coronavirus (COVID-19) pandemic broke out at the end of 2019 and has shown a trend of development worldwide. The spread of infectious disease rumors through social networks has been shown to cause public mood swings (Smith and Christakis, 2008; Hill et al., 2010) and can even affect people’s behavior, such as their cooperative behavior (Nowak and May, 1992; Ohtsuki et al., 2006). Rumors about infectious diseases will form an “emotional contagion” (Hatfield et al., 1994) in a short period of time and affect family relationships (Larson and Almeida, 1999), roommate relationships (Howes et al., 1985), and teammate relationships (Barsade, 2002), and even lead to large-scale emotional contagion via social networks (Kramer et al., 2014). This negative emotional contagion has been shown to cause significant economic damage. For example, overreaction of the government during the Southeast Asian respiratory syndrome led to a decline in the Asian tourism industry (Hai et al., 2004; McKercher and Chon, 2004), and fear and panic sentiments caused short-term damage to the Hong Kong economy (Siu and Wong, 2004). During the Southeast Asian crisis, some studies argued that the primary reason for the crisis was a sudden shift in market expectations and confidence (Feldstein, 1998; Radelet and Sachs, 1998; Stiglitz, 1999; Park and Song, 2001).

Emotional contagion also has a direct impact on professionals’ work emotions. Bartel and Saavedra (2000) studied the moods of 70 working groups and found that they could be divided into eight types of emotions and that the differentiation of work emotions is related to task and social interdependence, membership stability, and mood regulation norms, as well as others. Experiencing positive emotional contagion led to improved cooperation, decreased conflict, and increased perception of task performance (Barsade, 2002). Emotional contagion also affects a person’s social judgment (Doherty, 1998), affects leadership and job output (Johnson, 2008), affects gender differences (Doherty et al., 1995), and influences product attitudes (Howard and Gengler, 2001). Nofsinger (2005) argued that the general level of optimism or pessimism in society is reflected in the emotions of financial decision-makers. Social mood determines the types of decisions made by consumers, investors, and corporate managers alike. Extremes in social moods are characterized by optimistic (pessimistic) aggregate investment and business activity.

Most research on the impact of infectious diseases on the emotions is aimed, for the most part, at the public level and little attention is paid to the management community, especially financial managers. Emotional contagion of financial managers might be transmitted to financial markets and cause volatility. COVID-19 is a physical health threat to financial managers so it will also impact their investment decisions, which might further affect the volatility of financial markets. Financial managers are usually better at handling events involving risk than ordinary people. They also communicate through the industry community to make the most reasonable judgments regarding risks. There are two paths in this decision-making process: on the one hand, financial managers are worried about the impact of COVID-19 on their own organization’s business; on the other hand, they are also worried about their own relative competitiveness due to decision-making errors. By analyzing the occurrence mechanism of these two paths, it is helpful to understand how the risk of COVID-19 influences fluctuations in the financial market through the decision-making mechanism of financial managers. This paper investigates 422 financial managers in China and uses path analysis to explore the internal logic of the aforementioned decision-making mechanism.

## Method

The survey was conducted between February 23, 2020, and February 25, 2020, when the number of COVID-19 infections in China had not yet peaked and the disease had only just begun to spread globally, which led to increasing risks in the financial markets. Conducting a survey at this stage enabled us to obtain a more realistic perspective on financial managers’ perception of risks.

**FIGURE 1:**
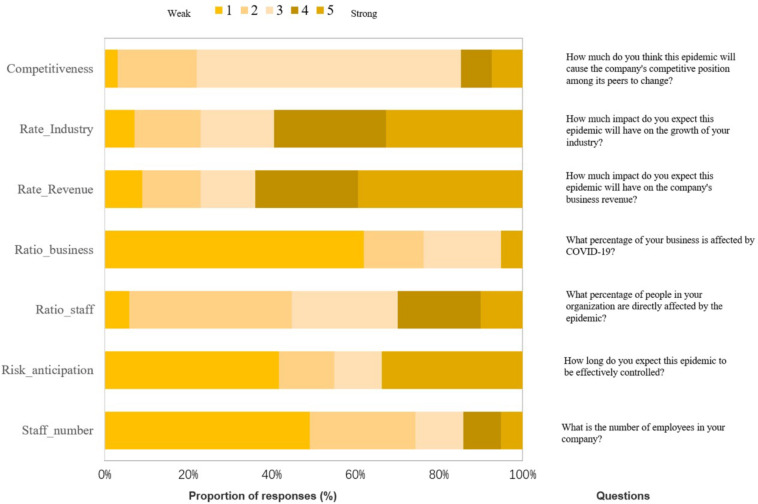
Questions for financial managers.

The survey was conducted using a questionnaire, which featured eight questions (see Figure 1). We first interviewed 10 managers by phone to learn about their judgments on the COVID-19 trend, the impacts on the company’s business, and the measures they took. Based on these interviews, we compiled an initial questionnaire and collected 30 samples. After analyzing the samples, we adjusted the questions and finally produced a questionnaire with eight questions. In the face of the COVID-19 outbreak, not all financial managers developed a comprehensive epidemic response strategy, so Question 8 was used to investigate whether they specifically developed a COVID-19 response plan (yes or no). The other seven questions were asked to investigate their risk perception (using a five-point scale). Based on the collected data, this paper used the path analysis model to analyze the financial managers’ decision-making logic regarding pandemic risk perception and in formulating their response strategy.

**FIGURE 2:**
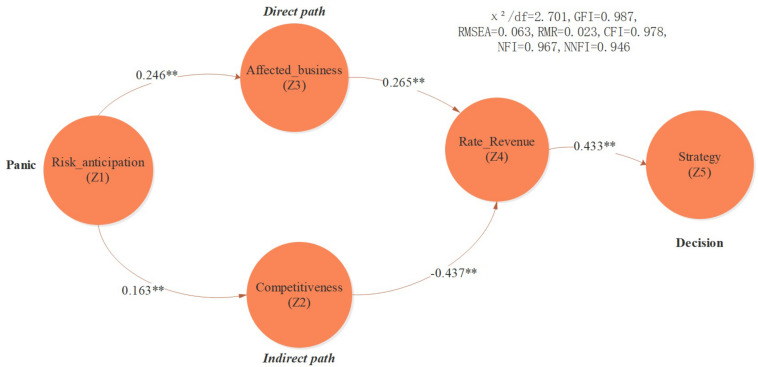
Path analysis results. Note: → indicates the path influence relationship, ^∗^*p* < 0.05, and ^∗∗^*p* < 0.01.

The path regression model is defined as (Figure 2):

$$Z_{2}=p_{12}\times Z_{1}$$

$$Z_{3}=p_{13}\times Z_{1}$$

$$Z_{4}=p_{24}\times Z_{2}+p_{34}\times Z_{3}$$

$$Z_{5}=p_{45}\times Z_{4}$$

*Z*_1_, *Z*_2_, *Z*_3_, *Z*_4_, *Z*_5_ represent decision variables, *p*_*ij*_ represents path coefficients.

## Results

The questionnaire was distributed randomly throughout associations in the financial industry, and a total of 422 valid responses were collected. The managers surveyed had more than 5 years of experience in the industry and often participated in events organized by industry associations. Among them, 64.93% of managers (*n* = 274) had previously formulated an outbreak response plan. Both the Kolmogorov–Smirnov test and the Shapiro–Wilk test were significant at a 1% level (*p* < 0.01), indicating that the data conformed to the characteristics of a normal distribution. In order to further analyze the financial manager’s decision-making mode when faced with epidemic-related risk, this paper used path analysis methodology to study the interrelationship between various factors. Figure 2 illustrates the path analysis chart and Table 1 demonstrates the path coefficients and fitting indicators. The MI values are far below 20 and the fitting indicators are also good which indicates that the path analysis model features good explanatory power.

$$\left(\begin{array}{c} \chi^{2}/d\,f=2.701,G\,F\,I=0.987,R\,M\,S\,E\,A=0.063,\\ R\,M\,R=0.023,C\,F\,I=0.978,N\,F\,I=0.967,N\,N\,F\,I=0.946 \end{array}\right),$$

**TABLE 1:** Regression—MI table.

*X*	→	*Y*	MI	Par change
Rate_Revenue	→	Affected _business	1.024	–0.214
Strategy	→	Affected_business	9.579	–0.372
Strategy	→	Rate_Revenue	3.313	0.403
Risk_anticipation	→	Rate_Revenue	1.024	0.051
Rate_Revenue	→	Competitiveness	1.024	0.332
Strategy	→	Competitiveness	0.130	–0.036
Affected_business	→	Strategy	8.942	–0.062
Competitiveness	→	Strategy	0.095	0.009
Risk_anticipation	→	Strategy	0.055	0.005
Rate_Revenue	→	Risk_anticipation	1.024	0.051
Strategy	→	Risk_anticipation	0.340	0.068

*→ indicates the path influence relationship.*

## Discussion

According to Figure 2, there are two significant paths that affect the manager’s decision-making process: the direct path and the indirect path. Faced with the uncertainty of COVID-19, whether a manager develops a coping strategy depends on their individual assessment of business revenue loss potential; their judgment is moderated by these direct and indirect effects. The direct effect refers to the risk perception directly caused by COVID-19, which is usually derived from the manager’s direct observations and risk expectations of infectious disease, by assessing the scope and duration of the epidemic’s spread. The indirect effect refers to managers worrying that they did not make timely adjustments or misjudged the situation, which might result in the loss of advantage amid fierce competition. Anxiety regarding the aforementioned two risks is the main reason that financial managers make decisions. The direct effect might cause managers to underrecognize or overreact to risks, while the indirect effect plays an intensification role, further contributing to managers’ panic.

Interestingly, the coefficient of the direct effect was 0.0652 (Risk_anticipation → Affected _business → Rate_Revenue), which was less than the coefficient (0.0712) of the indirect effect (Risk_anticipation → Competitiveness → Rate_Revenue), indicating that the indirect effect exceeded the direct effect. This might imply that financial managers are more rational than ordinary people when dealing with COVID-19, that they are more concerned about competitor dynamics, or that they might even view COVID-19 as an important opportunity to adjust their competitive position. This paper’s research results demonstrate that different communities feature significant differences in their perception of risk and behavioral patterns in terms of COVID-19. Evidence from financial managers can enable a better understanding of the potential impact of infectious disease risk on financial markets.

## Data Availability Statement

The datasets for this article are not publicly available because the data is only authorized for this study. Requests to access the datasets should be directed to the corresponding author, BC.

## Ethics Statement

The studies involving human participants were reviewed and approved by Central University of Finance and Economics. Written informed consent to participate in this study was provided by the participants.

## Author Contributions

JS was responsible for the overall research ideas, model design, and thesis writing. TW was responsible for document review writing and data collection and processing. BC was responsible for model optimization and discussion of research conclusions. All authors contributed to the article and approved the submitted version.

## Conflict of Interest

The authors declare that the research was conducted in the absence of any commercial or financial relationships that could be construed as a potential conflict of interest.
